# A horse’s locomotor signature: COP path determined by the individual limb

**DOI:** 10.1371/journal.pone.0167477

**Published:** 2017-02-14

**Authors:** Sandra Nauwelaerts, Sarah Jane Hobbs, Willem Back

**Affiliations:** 1 Department of Biology, University of Antwerp, Antwerp, Belgium; 2 Centre for Research and Conservation, Royal Zoological Society of Antwerp, Antwerp, Belgium; 3 Centre for Applied Sport and Exercise Sciences, University of Central Lancashire, Preston, United Kingdom; 4 Department of Equine Sciences, Faculty of Veterinary Medicine, Utrecht University, Utrecht, The Netherlands; 5 Department of Surgery and Anaesthesiology of Domestic Animals, Faculty of Veterinary Medicine, Ghent University, Merelbeke, Belgium; Szegedi Tudomanyegyetem, HUNGARY

## Abstract

**Introduction:**

Ground reaction forces in sound horses with asymmetric hooves show systematic differences in the horizontal braking force and relative timing of break-over. The Center Of Pressure (COP) path quantifies the dynamic load distribution under the hoof in a moving horse. The objective was to test whether anatomical asymmetry, quantified by the difference in dorsal wall angle between the left and right forelimbs, correlates with asymmetry in the COP path between these limbs. In addition, repeatability of the COP path was investigated.

**Methods:**

A larger group (n = 31) visually sound horses with various degree of dorsal hoof wall asymmetry trotted three times over a pressure mat. COP path was determined in a hoof-bound coordinate system. A relationship between correlations between left and right COP paths and degree of asymmetry was investigated.

**Results:**

Using a hoof-bound coordinate system made the COP path highly repeatable and unique for each limb. The craniocaudal patterns are usually highly correlated between left and right, but the mediolateral patterns are not. Some patterns were found between COP path and dorsal wall angle but asymmetry in dorsal wall angle did not necessarily result in asymmetry in COP path and the same could be stated for symmetry.

**Conclusion:**

This method is a highly sensitive method to quantify the net result of the interaction between all of the forces and torques that occur in the limb and its inertial properties. We argue that changes in motor control, muscle force, inertial properties, kinematics and kinetics can potentially be picked up at an early stage using this method and could therefore be used as an early detection method for changes in the musculoskeletal apparatus.

## Introduction

A certain degree of asymmetry between the left and right limb is considered normal [1]. In humans, anatomical asymmetry is linked to kinematic, kinetic and temporal gait asymmetries [2,3]; these functional differences reflect the mechanical compensations required to equilibrate limb length differences during locomotion [3] and are related to laterality [4]. In horses, skeletal asymmetries are found at a relatively high frequency [5]. Considerable anatomical asymmetry has been shown to negatively affect equine locomotor performance [6,7], and levels of functional asymmetry above predefined thresholds are used as a detection method for lameness in horses [8]. Nonetheless, there is quite a difference in perspective between horses owners and the veterinarians of their horses; more than a quarter of ‘owner-sound’ horse are considered to be lame by their veterinarian [9].

Multiple etiologies of acquired asymmetry have been described. Similar to humans, lateralization is also one of the suggested causes for horses. Foals with a limb preference during grazing develop more asymmetric feet [10]. Asymmetry can also occur due to training: left femurs of racehorses were found to be larger than right femurs, possibly due to asymmetric loading during left turns on a counter-clockwise racetrack [11]. However, preferential selection of racehorses with longer third metacarpal bones on their right limbs, enabling them to better negotiate counter-clockwise turns, might have occurred as well [12]. In addition, they may develop habitual postural or functional asymmetries due to pathological causes such as pain avoidance [13,14], neurological deficit [15], and/or hypoxia [16].

For humans with leg length inequalities, the shorter limb is loaded more and at higher rates [17]. Similarly, the side with the flatter foot is loaded more in horses [18]. Because the hoof is a dynamic structure that responds to its loading environment [19,20], hoof shape might be indicative for long-term loading and for loading distribution higher up the limb. As a proxy for hoof shape, we used dorsal hoof wall angle because hoof dimensions respond as one structure which makes foot width, heel angle and dorsal wall angles all interrelated [18,21,22].

The center of pressure (COP) position may be considered the net output variable of the interaction between all of the forces and torques that occur in the limb and its inertial properties, because it quantifies the dynamic load distribution under the hoof. In humans, this COP position over time, the COP path, provides insights into functional foot behaviour [23], and is highly symmetrical in normal gait [24]. In horses, pressure distribution has been studied, but analysis has usually been simplified to observational categorization [25], mean pressure in a region [26], contact area [27], or location of the COP in midstance [28], even though some authors used part of the COP path, during rolloff after midstance [29].

It remains unclear whether left-right differences in anatomy automatically lead to functional asymmetry in the way the horse moves. A recent study looking at ground reaction forces (GRF) in sound horses with asymmetric hooves found systematic differences in the horizontal braking force and relative timing of break-over [18]. In addition, the asymmetry between the two forelimbs proved to be more defining to the force changes than the individual conformation of each foot. We hypothesize that anatomical asymmetry, quantified by the difference in dorsal wall angle between the left and right forelimbs, correlates with asymmetry in the COP path between these limbs.

## Materials and methods

### Horses

31 horses of different breeds (24 Dutch Warmbloods, 1 Haflinger, 3 Arabians, 1 New Forest Pony, 1 Fjord, 1 Friesian, see Table 1), masses (mean: 551 kg ± 79) and ages (mean: 11 years) of age ± 6 participated in this study. 20 horses were shod and 11 were barefoot. All horses were either client-owned, in which case the owners consented to the study, or they were school horses. This study was conducted in accordance with Dutch Law.

**Table 1:** Mean values per horse per limb. Each horse is represented by one row, age, breed, total mass and shod or unshod are indicated, the shape of the COP path for both left (L) and right (R) limb, relative COP position at footstrike, midstance and lift-off.

				Dorsal Wall Angle		Shape		Footstrike R		Midstance R		Lift off R		Footstrike L		Midstance L		Lift off L	
Age	Mass (kg)	Breed	Shoeing	L	R	L	R	COPx	COPy	COPx	COPy	COPx	COPy	COPx	COPy	COPx	COPy	COPx	COPy
9	355	New Forest	shod	41	54	n	u	-0.26	0.24	-0.03	0.15	0.15	0.22	-0.22	0.18	-0.17	0.11	-0.05	0.08
10	536	Friesian	shod	53	49	n	u	0.35	0.13	0.14	-0.03	0.06	0.18	0.13	-0.03	0.01	0.00	-0.04	0.08
4	567	KWPN	shod	51	52	ℓ	ⱴ	0.29	0.13	0.11	0.03	0.11	0.19	0.31	0.05	-0.02	-0.01	0.00	0.18
6	498	KWPN	unshod	50	52	L	ℓ	0.31	-0.05	0.09	-0.12	0.08	0.15	0.37	-0.28	0.03	-0.13	0.01	0.16
5	484	Arabian	shod	52	54	8	8	0.10	0.06	0.05	-0.06	0.09	-0.07	0.18	0.25	0.02	0.09	-0.02	-0.01
4	584	KWPN	shod	57	49	ⱴ	ⱴ	0.31	-0.15	0.12	-0.28	0.05	-0.15	0.19	-0.01	-0.15	0.05	0.02	0.12
27	549	KWPN	shod	48	46	♥	ℓ	0.38	-0.20	0.17	-0.05	0.15	0.18	-0.10	0.17	-0.08	-0.02	-0.02	0.16
3	541	KWPN	unshod	52	55	L	ⱴ	0.42	-0.08	0.17	0.04	0.11	0.07	0.39	-0.08	0.02	-0.02	0.07	0.08
9	540	KWPN	shod	54	55	U	ℓ	0.24	0.03	-0.02	-0.05	0.04	0.12	-0.12	0.04	-0.05	-0.05	0.05	0.16
2	449	KWPN	unshod	54	62	u	8	0.12	0.08	0.04	-0.04	0.05	0.11	0.19	0.12	-0.10	-0.11	-0.09	0.11
10	424	Arabian	shod	50	50	u	8	0.05	0.20	0.06	0.06	0.14	0.01	0.26	0.20	0.04	0.08	0.00	0.04
19	525	KWPN	shod	47	50	ⱴ	ℓ	0.27	0.04	0.08	-0.07	0.11	0.10	0.06	0.19	-0.05	-0.06	0.05	0.09
18	572	KWPN	unshod	59	51	ⱴ	ℓ	0.32	0.19	0.02	0.16	0.06	0.26	0.29	0.36	-0.05	0.03	-0.06	0.19
9	438	Fjord	shod	52	52	♥	8	0.42	-0.06	0.14	0.04	0.16	0.15	-0.15	0.10	-0.10	0.03	0.03	0.18
18	670	KWPN	unshod	47	57	ϑ	ℓ	0.36	-0.17	0.09	-0.06	0.02	0.21	-0.12	0.28	-0.02	0.07	-0.07	0.09
17	578	KWPN	unshod	52	47	∫	ζ	0.35	-0.27	0.08	-0.06	0.19	0.26	0.08	-0.10	-0.14	-0.05	0.04	0.24
15	591	KWPN	shod	49	49	∫	ℓ	0.42	-0.02	0.18	-0.05	0.23	0.10	-0.23	-0.18	-0.11	-0.02	-0.02	0.17
16	514	KWPN	shod	49	47	U	ℓ	0.37	0.04	0.15	-0.03	0.10	0.21	0.16	0.14	-0.03	-0.02	-0.01	0.17
19	557	KWPN	unshod	49	54	ϑ	L	0.26	0.02	0.08	-0.04	0.08	0.19	-0.16	0.27	-0.11	-0.05	-0.02	0.15
14	690	KWPN	unshod	50	54	ℓ	ζ	0.36	-0.10	0.07	-0.04	0.05	0.08	0.12	0.18	-0.05	0.10	-0.01	0.16
14	616	KWPN	shod	66	69	ℓ	ℓ	0.45	0.19	0.18	0.08	0.19	0.19	0.30	0.01	-0.05	0.10	-0.08	0.25
12	568	Arabian	unshod	44	61	ℓ	ζ	-0.05	-0.29	0.02	0.01	0.07	0.22	0.06	-0.15	-0.05	-0.06	-0.03	0.09
11	641	KWPN	shod	49	44	ℓ	ȷ	0.36	0.21	0.02	-0.05	-0.02	0.16	0.34	0.14	0.02	-0.04	0.00	0.21
10	690	KWPN	shod	50	48	8	Ʊ	0.38	0.06	0.10	-0.02	0.09	0.10	0.17	-0.06	-0.03	-0.02	0.02	0.07
11	620	KWPN	shod	54	50	♥	8	0.14	0.11	0.04	-0.05	0.08	0.11	0.08	0.19	-0.06	-0.02	0.04	0.12
9	492	KWPN	shod	50	54	ⱴ	ⱴ	0.31	-0.22	0.05	0.01	0.04	0.18	0.08	-0.02	-0.02	0.03	-0.04	0.15
11	653	KWPN	shod	47	41	8	8	0.06	0.36	0.02	0.04	0.03	0.14	0.11	0.18	-0.06	-0.02	-0.02	0.12
10	593	KWPN	unshod	57	58	ⱴ	8	0.25	0.09	0.08	0.06	0.08	0.23	0.11	0.11	-0.02	0.01	-0.01	0.11
7	590	KWPN	shod	48	50	ℓ	ℓ	0.32	0.12	0.10	-0.02	0.12	-0.01	0.29	0.08	-0.04	-0.06	-0.03	0.02
10	479	Haflinger	unshod	58	51	ℓ	ℓ	0.31	-0.28	0.11	-0.09	0.08	0.06	0.25	-0.22	0.05	0.05	-0.02	0.19
8	493	KWPN	shod	46	49	ℓ	ȷ	-0.09	0.02	0.00	0.00	0.07	0.16	0.17	0.07	0.03	-0.02	0.07	0.22

### Data collection

Hoof kinematics and pressures underneath the hooves were measured simultaneously. Hoof kinematics were observed using a Qualisys motion analysis system consisting of 8 high-speed Oqus 3+ cameras recording at 250 Hz. Pressures underneath the hooves were measured using the Gait Scientific Software capturing data from a Footscan 3-D 1 m-system measuring at 125 Hz (RsScan International). Video data were synchronized with the pressure data.

Prior to the trotting experiments, 4 markers were attached to the hooves of the forelimbs of each horse. Two markers defining the mediolateral midline of the hoof. One marker in front to define the dorsopalmar midline, perpendicular on the mediolateral line. This marker defined together with a fourth, more proximal marker the dorsal wall angle (see Data Analysis).

Photographs were taken from underneath to correct for possible misplacement of the three lower markers during the experiments (see Data Analysis). A standing trial with the horse standing still was captured prior to the moving trials.

A handler led each horse at trot in a straight line through the capture volume. The trial started 5 meters before the pressure mat and the horse was encouraged to trot at a consistent velocity for approximately 5 meters past the capture volume (i.e. over the entire length of the rubber mat). Three measurements per foot with hits on the pressure mat were recorded. Trials selected for further analysis met the following criteria: they obtained the pressure underneath the forelimb during an entire stance phase, the horse moved at a steady speed, and the markers on its hooves were present for the complete stance phase.

### Data processing

Marker positions during the standing trial were used to define the dorsal wall angle for each of the forelimbs. The angle was measured in the sagittal plane of the hoof as the angle (from the horizontal) between the distal (or lower) dorsal hoof marker and the proximal dorsal hoof marker.

For the running trials, a local coordinate system was created based on the positions of the three distal markers. The X-axis was oriented from the medial to the lateral marker. The Y-axis was oriented perpendicular on the X-axis and through the frontal marker. This coordinate system was drawn on the pictures taken prior to the experiments, together with a coordinate system based on the hoof. An ellipse was drawn outlining the hoof wall, and the minor and major axes of this ellipse were used as the true local hoof-bound coordinate system. The difference between the two coordinate systems was quantified by an angle of rotation and two translations. These correction factors were used to correct for marker displacement in the standardization of the COP path.

COP position in X and Y was calculated first in the Footscan coordinate system using a custom written Matlab program (Mathworks, v.8.4 2014) by taking the weighted average of the pressures, knowing their position and sensor size. The coordinate system of the pressure mat was lined up with the coordinate system of the kinematic data in both position and time (accounting for the 20 ms delay). For each frame during the stance phase, a local coordinate system (based on the 3 distal markers on the hoof) was created and the COP path was calculated in this system. This method, therefore, moved with the hoof and slip was removed from the COP path throughout stance. For each trial, correction factors were applied to ensure the origin of the local coordinate system was in the middle of the hoof. To start, COP variables were calculated as absolute values. Then positions at touchdown, midstance and lift-off were determined, together with the amplitudes along both axes. Finally, using the hoof dimensions, COP data were normalized to hoof width in the mediolateral direction and to hoof length in the dorsopalmar direction.

### Data analysis

For each trial, COP paths, for both raw data and data recalculated in the hoof-bound coordinate system, were time normalized to 101 points using a spline interpolation routine in Matlab. These 101 datapoints were loaded into MorphoJ, software to analyze shapes [30]. After a Procrustes superimposition, in which shape and size are extracted from the data, a Procrustes ANOVA was performed using horse and side as fixed effects and including the interaction in the model. Repeatability was calculated based on the sum of squares from both centroid size and Procrustes shape.

All statistical tests were performed in SPSS Statistics 20 (IBM Corp.). A paired t-test was used to test for systematic differences between left and right dorsal wall angle. To determine the correlation in COP path between left and right, three normalised trials on each limb were averaged and the standard deviation determined. Correlations between COP paths of left and right were obtained in two dimensions. These correlations quantified the degree of functional asymmetry. A link between functional asymmetry and anatomical asymmetry was tested for by regressing the difference in dorsal wall angle against the two correlations of the left-right COP path. Correlations between the 2D distances from each data point to the line y = x (perfect symmetry) for each COP variable and the 2D distances from each data point to the line y = x of the dorsal wall angle were evaluated. A link between COP path and dorsal wall angle was tested for each hoof by a MANCOVA using limb and horse as fixed effects and dorsal wall angle as a covariate. All COP variables were put into this model. Categories were made based on COP path shape (see Results) and dorsal wall angle was compared between the different categories using a one-way ANOVA. To test for codependence between the COP variables, a PCA was performed.

## Results

**Fig 1:**
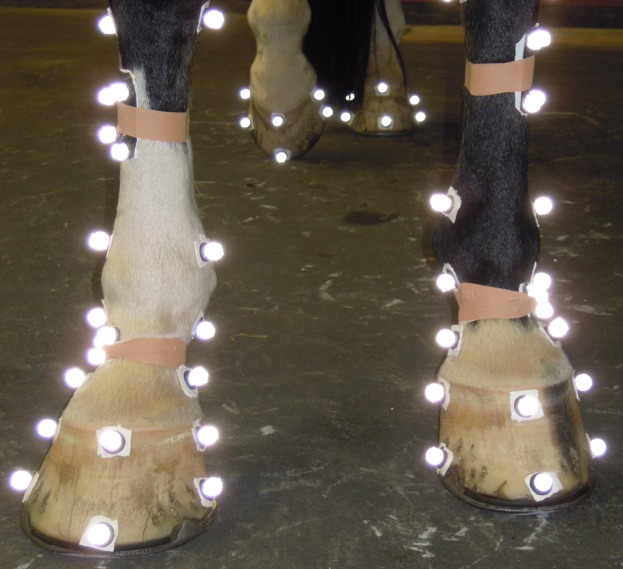
Marker setup of both limbs. Joint centers of the lower limb and 6 markers on each hoof were attached to the horse using sticky tape.

Repeatability of the COP pattern in the hoof-bound coordinate system is high, as demonstrated in the example file in Fig 1. Repeatabilities for centroid size (the size of the COP path) was 0.79, for shape 0.35. This was due to a large individual effect on size and shape as well (p<0.0001) as the asymmetry in the COP path between left and right (p<0.0001). COP paths still expressed in the global coordinate system were not repeatable: repeatabilities dropped to 0.22 (centroid size) and 0.24 (shape).

### COP variables

**Fig 2:**
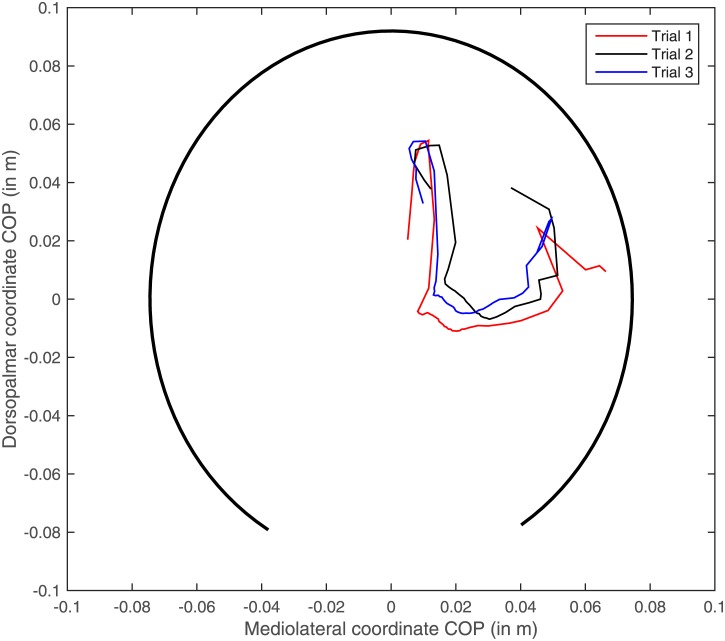
Example file of the center of pressure (COP) path of three trials from the same limb. COP coordinates are colour coded red, black and blue. The thick black line shows a partial ellipse drawn based on the hoof dimension of this limb to demonstrate relative position of the COP path in a hoof-bound coordinate system.

A lateral landing was defined as a mediolateral COP greater than zero (with zero being the middle of the hoof). From the 222 trials from 32 horses, we found that 175/222 landed laterally and 145/222 landed in front of the center of the hoof, towards the toe. In midstance, for only 35 trials, the COP position was close (within 2%) to the center in the mediolateral direction and 46 in the dorsopalmar direction, with just over half (126/222) with a COP position in the lateral half of the hoof and 125/222 in the caudal half. Mean relative amplitude was 31 ± 11% in the mediolateral direction, and 40 ± 13% in the craniocaudally direction, Table 1. Different shapes of COP patterns were detected (L, mirrored L, 8, U, reversed U, heart-shaped; see Fig 2 for examples, Table 1).

### Left-right asymmetry

**Fig 3:**
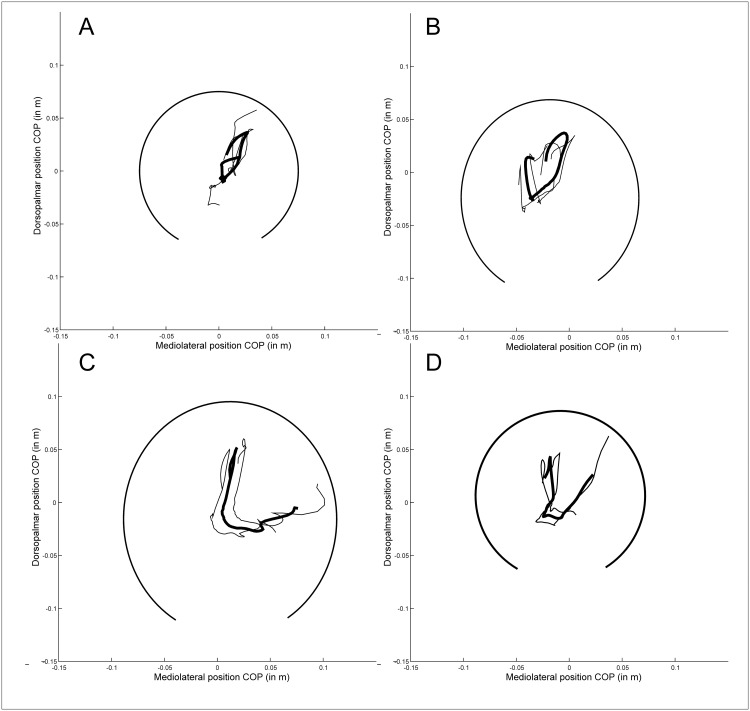
Example files of different COP path shapes. The thick line shows the mean COP path over three trials for one limb of one individual, while the thinner lines show the mean ± standard deviation. The thick black line shows a partial ellipse drawn based on the hoof dimension of this limb to demonstrate relative position of the COP path in a hoof-bound coordinate system. A. 8-shape, B. heart shape, C. L-shape and D. U-shape.

COP path in the fore-aft direction has a high correlation *r* between left and right: in 28 of the 31 cases, *r* was larger than 0.85. The three exceptions have a lateral landing in the middle of the hoof on the right and a medial toe landing on the left, with the rolloff being similar between left and right (Fig 3).

**Fig 4:**
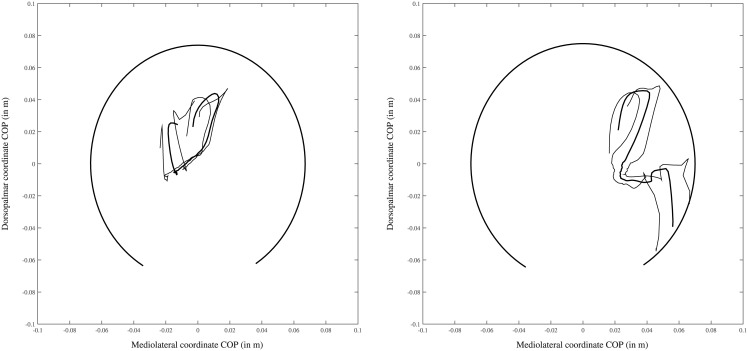
Example file of one of the three “exception” horses in which the correlation in the fore-aft direction is low. The thick line shows the mean COP path over three trials, while the thinner lines show the mean ± standard deviation. The thick black line shows a partial ellipse drawn based on the hoof dimension of this limb to demonstrate relative position of the COP path in a hoof-bound coordinate system.

**Fig 5:**
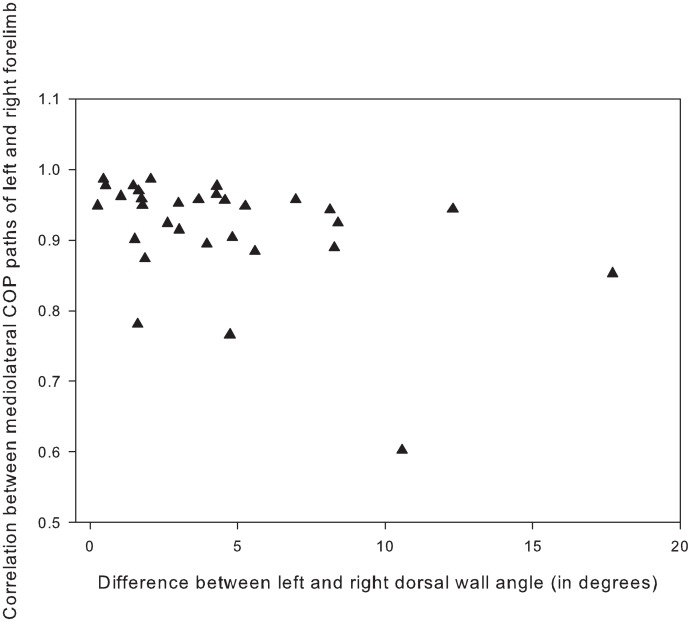
Correlation between the mediolateral paths and difference in dorsal wall angle of left and right forelimbs. Mediolateral paths plotted against the absolute value of the difference in dorsal wall angle of the hooves of both forelimbs. Note the large variation in *r* and the lack of correlation with the asymmetry of the hoof anatomy.

COP path in the mediolateral direction has a wide range of correlations between left and right, but no clear pattern was found between COP asymmetry and hoof asymmetry (Fig 4). 9/31 horses have mirrored COP paths with high correlations (*r* between 0.85 and 0.98, mean 0.91 ± 0.04), of which some have large differences in dorsal wall angle between left and right (Fig 5), but overall the dorsal wall differences for this group are between 1 and 8 degrees, with a mean difference of 4.2 ± 2.2 degrees. 4/31 horses have the same pattern (not mirrored) on both sides (high negative correlations between -0.68 and -0.49, mean -0.56 ± 0.08), of which two are completely symmetrical (difference in dorsal wall angle zero), and two are very asymmetrical (difference in dorsal wall angle 11 and 5 degrees). The largest group, however, (12/31) has a moderately correlated mirrored COP path (*r* between 0.46 and 0.84, mean 0.64 ± 0.13), of which most are asymmetrical (mean difference in dorsal wall angle is 5.5 degrees ± 5.1). For 6/31 horses, the COP paths were uncorrelated (*r* between -0.22 and 0.31, mean 0 ± 0.19). In this group, the difference in dorsal wall angle differed between 1 and 8 degrees, with a mean of 3.3 ± 2.5 degrees.

**Fig 6:**
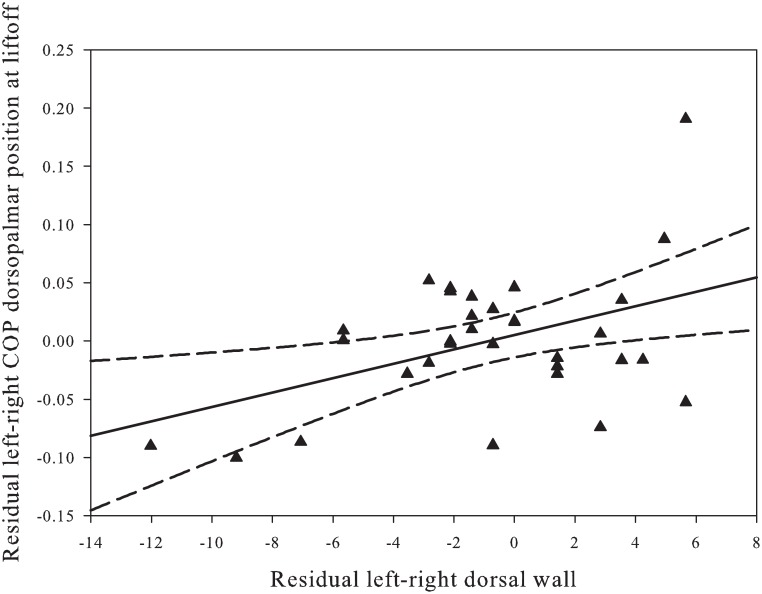
Residual analysis of COP position at lift off and dorsal hoof wall angle. 2-D distance of the COP position at lift-off in the dorsopalmar direction to the left equals right equation is correlated to the 2-D distance of the dorsal wall angle of right plotted against left. Note that the more the positions differ in the two forelimbs, the more the COP position also differs.

Correlations between the residuals of left and right in the COP variables and the residuals in dorsal wall angle show a significant correlation between the craniocaudal position of the COP at lift-off and the dorsal wall angle (Fig 6).

### Link between COP path and hoof dorsal wall angle

On average, the right limb has a larger dorsal wall angle than the left limb (52.9 ± 5.8 vs. 50.9 ± 4.5 degrees). However, the outcome of a paired *t*-test showed that this is not a significant difference (*p* 2-tailed is 0.272).

COP pattern is highly dependent on the horse, except for the amplitude in the mediolateral direction (*p* = 0.075). Differences between left and right are also horse-dependent, being significant for all mediolateral COP variables. At touchdown the COP lands on average 16 mm (± 3 mm) more laterally in the right limb, and stays more lateral in midstance with the COP being 13 mm lateral from the center in the right hoof; conversely, the COP is 7 mm medial from the center in the left hoof and ends in a 12 mm more lateral position at lift-off. This results in an 11 mm larger amplitude in the left limb compared to the right. In the craniocaudal direction, only position of the COP in midstance differs significantly (but slightly) between left and right, with COP being 2 mm off center in the posterior direction on the right while being exactly in the middle on the left.

Both relative and absolute data yield the same results. There was a significant correlation between the COP position in midstance (*p* = 0.043), amplitude (*p* = 0.003) both in the mediolateral direction, and the dorsal wall angle. PCA on all COP variables yielded three groups: (1) COP positions in the mediolateral direction at three moments during stance, (2) both amplitudes, negatively correlated with position in the craniocaudal direction, and (3) COP position in the craniocaudal direction at midstance and liftoff. None of the PCA scores were correlated with dorsal wall angle, and all COP factors were horse-dependent. Factor 1 is different between left and right limbs.

Hoof angles did not differ significantly between the COP path shape categories.

## Discussion

Our dataset consisted of dorsal angles between 41 and 69 degrees. Forelimbs have been reported to ideally have angles between 45 and 50 [31], but experimental work has reported mean angles of around 50 [32,33]. This means our data included “normal” hoof angles as well as low (flat) and steep (upright) ones.

The procedure of combining the COP data from the pressure plate and the instantaneous hoof location from motion capture yielded highly repeatable data. This reduction in variation compared to raw COP data in a global, stationary coordinate system is probably due to subtraction of “slide” of the hoof. Knowing the exact location of the COP with respect to the hoof is important because this relative position determines the accuracy of the ground reaction vector with respect to the entire limb. Previous studies have either not taken this into account [26] or manually fitted a picture of the hoof on top of the pressure profile at midstance [28] rather than using an independent, synchronized camera system that locates the hoof throughout the entire stance phase.

Theoretically, the “ideal” COP path for a forelimb, which is a straight leg, would achieve three goals during locomotion: during impact, it would be in the ideal position to enable heel expansion, dampening in the digital cushion and dampening of the vibrations from the impact shock by the muscles higher up the limb. At midstance, the expectation is that the COP position will be optimized according to a minimization of all the joint moment arms. This minimization would ensure the most efficient force input-to-output ratio [34]. This most likely means that, mediolaterally, the COP would be close to the middle of hoof in order for the ground reaction force to be in the midsaggital plane of the limb; craniocaudally, we would expect it to be close to the projection of the elbow joint position to the ground. Even though the hoof wall has the ability to prevent the formation of cracks, during breakover strain in the hoof briefly increases [35]. In this end phase of stance, the COP would probably be chosen to minimize bending moments on the hoof wall. If so, one would expect the COP to be close to the wall and the GRF parallel to it. However, despite these theoretical considerations, one must realize that the COP position is the outcome of multiple processes interacting which each other: these include, but are not limited to anatomy of the joints and segments, segment inertial properties, motor patterns, force and moment patterns, kinematics and the hoof-surface interaction. The COP paths are repeatable, but in spite of this it might be hard to theorize on where they should be. In fact, we might philosophize that ideal COP paths do not exist, but that the resulting path is the result of multiple optimization processes happening simultaneously and is the best possible solution for this individual limb.

Interpreting the COP path as the best possible outcome of multiple opposing optimization mechanisms might explain why in these sound horses the COP paths are very consistent but rarely perfectly mirrored between left and right. The craniocaudal patterns are usually highly correlated, but the mediolateral patterns are not. Granted, such nicely mirrored paths exists, but sometimes they occur in asymmetric horses, and even in the horses we defined as being symmetrical low correlations between left and right occur. This might be due to our definition of asymmetry which is solely based on the dorsal wall angle, even though this parameter in itself can also be interpreted as an end result of the dynamics occurring higher up the limb. Even so, our interpretation is that the motor control of each limb and its dynamics has been shaped in such a way that the limb moves optimally, rather than the objective of the motor control being to keep the motor patterns symmetrical; pain and/or hypoxia is then interfering with these biomechanically and neurologically predefined locomotor patterns.

Reasons other than differences in dorsal wall angles for why patterns deviate from perfectly mirrored COP paths are that limb placement with respect to the COM (centre of mass) might be different between left and right; the flight arc of the hoof prior to contact might be different; and the slip distance might be different.

We also found systematic differences between left and right across all horses (symmetric and asymmetric together). It has been reported that left hooves are usually larger [36,37], but even when this is corrected for the differences remain (albeit a slight shift). This systematic difference might be due to laterality, differences in conformation other than dorsal wall angle, difference in limb strength or muscle development, and (as mentioned above) differences in limb placement.

## Conclusions

The use of a local coordinate system that moves with the hoof (removing the effect of sliding forward) makes COP path very repeatable, unlike in previous studies. Many shapes of COP paths are possible in sound horses, but they are unique to the limb and the individual. We could not detect any clear patterns of COP path shape with dorsal wall angle. Since the COP path is the net result of numerous variables—conformation, kinematics, dynamics, hoof-substrate interaction, and muscle physiology (muscle co-contractions, muscle strength, etc)—it is hard to theoretically predict what the optimal COP path for a given individual would be. However, because of the consistency in the patterns, we suggest this method is so sensitive it could provide an early detection method for musculoskeletal issues when COP path shape is followed up through time. We expect that rather than comparing the patterns to an average norm, comparing the patterns to an individual baseline for the individual limb, and taken natural fluctuations due to hoof growth into account, will enable the detection of subtle changes in the system. However, the pattern of change over time still needs to be investigated to ensure the repeatability of the COP path remains as high over time as it was within one day. If it is not repeatable, the technique might still be applicable if the changes over time themselves are repeatable. To test this idea, a longitudinal study in which a number of horses are measured frequently for a long period of time is required. During such a study, it might be possible to retrospectively look at horses with increasing lameness to test whether the changes are detectable at an early stage. Encouraging in that respect is the study that found differences in COP path between sound horses and horses with navicular disease [38].
